# *N*′^2^,*N*′^5^-Bis[(*E*)-2-hy­droxy­benzyl­idene]-3,4-dimethyl­thio­phene-2,5-dicarbohydrazide

**DOI:** 10.1107/S1600536812020260

**Published:** 2012-05-16

**Authors:** Shao-Lin Zhang, Ling Zhang, Qin-Mei Wen, Rong-Xia Geng, Cheng-He Zhou

**Affiliations:** a Laboratory of Bioorganic & Medicinal Chemistry, School of Chemistry and Chemical Engineering, Southwest University, Chongqing 400715, People’s Republic of China

## Abstract

In the title mol­ecule, C_22_H_20_N_4_O_4_S, both C=N bonds are in an *E* conformation. The benzene rings form dihedral angles of 12.10 (13) and 25.17 (12)° with the thio­phene ring. The dihedral angle between the two benzene rings is 17.59 (14)°. There are two intra­molecular O—H⋯N hydrogen bonds. In the crystal, N—H⋯O hydrogen bonds connect mol­ecules into chains along [010].

## Related literature

For the medicinal properties of thio­phene derivatives, see: Bondock *et al.* (2010 ▶); Geng & Zhou (2008 ▶). For a related structure, see: Tang *et al.* (2010 ▶).
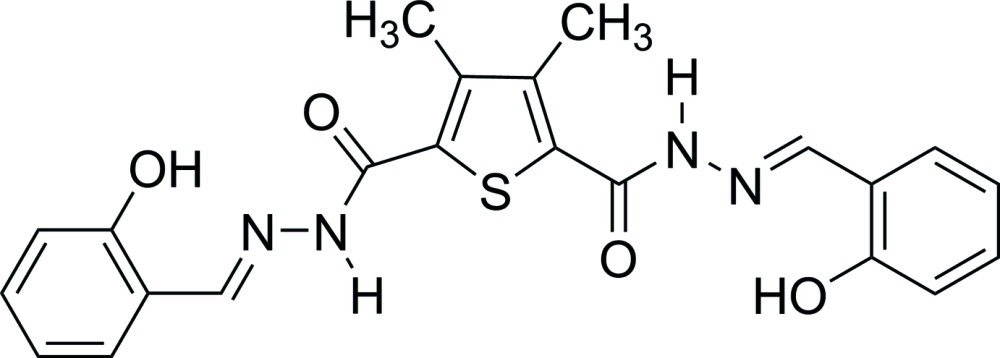


## Experimental

### 

#### Crystal data

C_22_H_20_N_4_O_4_S
*M* *_r_* = 436.48
Triclinic, 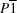

*a* = 8.392 (8) Å
*b* = 9.511 (9) Å
*c* = 12.937 (8) Å
α = 99.853 (17)°
β = 90.804 (18)°
γ = 92.374 (18)°
*V* = 1016.2 (15) Å^3^
*Z* = 2
Mo *K*α radiation
μ = 0.20 mm^−1^
*T* = 296 K
0.19 × 0.18 × 0.16 mm

#### Data collection

Bruker APEXII CCD diffractometer
Absorption correction: multi-scan (*SADABS*; Bruker, 2009 ▶) *T*_min_ = 0.963, *T*_max_ = 0.969
5573 measured reflections
3925 independent reflections
2660 reflections with *I* > 2σ(*I*)
*R*_int_ = 0.019

#### Refinement

*R*[*F* ^2^ > 2σ(*F* ^2^)] = 0.047
*wR*(*F* ^2^) = 0.128
*S* = 1.02
3925 reflections
283 parameters
H-atom parameters constrained
Δρ_max_ = 0.23 e Å^−3^
Δρ_min_ = −0.30 e Å^−3^

### 

Data collection: *APEX2* (Bruker, 2009 ▶); cell refinement: *SAINT* (Bruker, 2009 ▶); data reduction: *SAINT*; program(s) used to solve structure: *SHELXS97* (Sheldrick, 2008 ▶); program(s) used to refine structure: *SHELXL97* (Sheldrick, 2008 ▶); molecular graphics: *SHELXTL* (Sheldrick, 2008 ▶); software used to prepare material for publication: *SHELXTL*.

## Supplementary Material

Crystal structure: contains datablock(s) global, I. DOI: 10.1107/S1600536812020260/lh5463sup1.cif

Structure factors: contains datablock(s) I. DOI: 10.1107/S1600536812020260/lh5463Isup2.hkl

Supplementary material file. DOI: 10.1107/S1600536812020260/lh5463Isup3.cml

Additional supplementary materials:  crystallographic information; 3D view; checkCIF report

## Figures and Tables

**Table 1:** Hydrogen-bond geometry (Å, °)

*D*—H⋯*A*	*D*—H	H⋯*A*	*D*⋯*A*	*D*—H⋯*A*
O1—H1⋯N1	0.82	1.91	2.626 (4)	145
N2—H2⋯O3^i^	0.86	1.97	2.789 (4)	159
N4—H4⋯O2^ii^	0.86	1.97	2.812 (4)	165
O4—H4*B*⋯N3	0.82	1.85	2.569 (4)	146

Symmetry codes: (i) 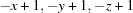; (ii) 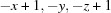.

**Fig. 1.**
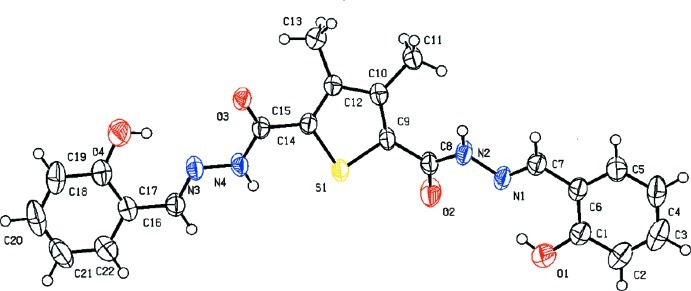
The molecular structure of (I), showing the displacement ellipsoids drawn at the 50% probability level.

## supplementary crystallographic information

### Comment

Thiophene is a electron-rich five-membered aromatic heterocycle containing a sulfur atom whose derivatives display various biological activities. Much effort has been devoted to the researches of thiophene-based compounds as medicinal agents (Geng *et al.*, 2008; Bondock *et al.*, 2010). Our interest is to develop novel thiophene compounds with high bioactivities especially broad antimicrobial spectrum. We have already prepared a thiophene compound incorporating Schiff base moieties and determined its crystal structure (Tang *et al.*, 2010). Herein, the crystal structure of title compound (I) is reported.

The molecular structure of (I) is shown in Fig. 1. Both C═N bonds are in an E conformation. The benzene rings form dihedral angles of 12.10 (13)° (C17-C22) and 25.17 (12) ° (C1-C6) with the thiophene ring (S1/C9/C10/C12/C14). The dihedral angle between the two benzene rings is 17.59 (14)°. There are two intramolecular O—H···N hydrogen bonds. In the crystal, N—H···O hydrogen bonds connect molecules into chains along [010].

### Experimental

A mixture of 3,4-dimethylthiophene-2,5-dicarbohydrazide (0.11 g, 0.5 mmol) and 2-hydroxybenzaldehyde (0.24 g, 2 mmol) in methanol (10.0 ml) was stirred at room temperature. Upon the completion of the reaction (monitored by TLC, eluent, ethyl acetate), the formed precipitate was filtered and then washed with cold methanol to afford a yellow solid of the title compound (0.35 g). A crystal suitable for X-ray analysis was grown from a mixed solution of (I) in chloroform and methanol by slow evaporation at room temperature.

### Refinement

H atoms were placed in calculated positions with C—H = 0.93 Å (aromatic), 0.96 Å (methyl), N—H = 0.86Å and O—H = 0.82Å. The *U*_iso_(H) values were set equal to 1.2U_eq_(C_aromatic_,N) and 1.5U_eq_(C_methyl_,O).

### Crystal data

**Table:**

C_22_H_20_N_4_O_4_S	*Z* = 2
*M**_r_* = 436.48	*F*(000) = 456
Triclinic, *P*1	*D*_x_ = 1.426 Mg m^−^^3^
Hall symbol: -P 1	Mo *K*α radiation, λ = 0.71073 Å
*a* = 8.392 (8) Å	Cell parameters from 1480 reflections
*b* = 9.511 (9) Å	θ = 2.9–24.4°
*c* = 12.937 (8) Å	µ = 0.20 mm^−^^1^
α = 99.853 (17)°	*T* = 296 K
β = 90.804 (18)°	Block, yellow
γ = 92.374 (18)°	0.19 × 0.18 × 0.16 mm
*V* = 1016.2 (15) Å^3^	

### Data collection

**Table:**

Bruker APEXII CCD diffractometer	3925 independent reflections
Radiation source: fine-focus sealed tube	2660 reflections with *I* > 2σ(*I*)
Graphite monochromator	*R*_int_ = 0.019
φ and ω scans	θ_max_ = 26.0°, θ_min_ = 2.4°
Absorption correction: multi-scan (*SADABS*; Bruker, 2009)	*h* = −9→10
*T*_min_ = 0.963, *T*_max_ = 0.969	*k* = −11→10
5573 measured reflections	*l* = −15→15

### Refinement

**Table:**

Refinement on *F*^2^	Secondary atom site location: difference Fourier map
Least-squares matrix: full	Hydrogen site location: inferred from neighbouring sites
*R*[*F*^2^ > 2σ(*F*^2^)] = 0.047	H-atom parameters constrained
*wR*(*F*^2^) = 0.128	*w* = 1/[σ^2^(*F*_o_^2^) + (0.0604*P*)^2^ + 0.0881*P*] where *P* = (*F*_o_^2^ + 2*F*_c_^2^)/3
*S* = 1.02	(Δ/σ)_max_ < 0.001
3925 reflections	Δρ_max_ = 0.23 e Å^−^^3^
283 parameters	Δρ_min_ = −0.30 e Å^−^^3^
0 restraints	Extinction correction: *SHELXL97* (Sheldrick, 2008), Fc^*^=kFc[1+0.001xFc^2^λ^3^/sin(2θ)]^-1/4^
Primary atom site location: structure-invariant direct methods	Extinction coefficient: 0.0062 (17)

### Special details

**Table:**

Geometry. All e.s.d.'s (except the e.s.d. in the dihedral angle between two l.s. planes) are estimated using the full covariance matrix. The cell e.s.d.'s are taken into account individually in the estimation of e.s.d.'s in distances, angles and torsion angles; correlations between e.s.d.'s in cell parameters are only used when they are defined by crystal symmetry. An approximate (isotropic) treatment of cell e.s.d.'s is used for estimating e.s.d.'s involving l.s. planes.
Refinement. Refinement of *F*^2^ against ALL reflections. The weighted *R*-factor *wR* and goodness of fit *S* are based on *F*^2^, conventional *R*-factors *R* are based on *F*, with *F* set to zero for negative *F*^2^. The threshold expression of *F*^2^ > σ(*F*^2^) is used only for calculating *R*-factors(gt) *etc*. and is not relevant to the choice of reflections for refinement. *R*-factors based on *F*^2^ are statistically about twice as large as those based on *F*, and *R*- factors based on ALL data will be even larger.

### Fractional atomic coordinates and isotropic or equivalent isotropic displacement parameters (Å^2^)

**Table:**

	*x*	*y*	*z*	*U*_iso_*/*U*_eq_	
N1	0.9005 (2)	0.2862 (2)	0.73465 (15)	0.0457 (5)	
N2	0.8176 (2)	0.32288 (19)	0.65213 (15)	0.0463 (6)	
H2	0.8112	0.4109	0.6455	0.056*	
N3	0.0145 (2)	0.15155 (19)	0.30284 (15)	0.0423 (5)	
N4	0.1633 (2)	0.16130 (19)	0.34807 (16)	0.0448 (5)	
H4	0.1990	0.0926	0.3759	0.054*	
C1	1.0429 (3)	0.2314 (3)	0.92331 (19)	0.0473 (6)	
C2	1.1287 (4)	0.2087 (3)	1.0091 (2)	0.0631 (8)	
H2A	1.1110	0.1251	1.0363	0.076*	
C3	1.2397 (4)	0.3085 (4)	1.0542 (2)	0.0702 (9)	
H3A	1.2992	0.2919	1.1118	0.084*	
C4	1.2661 (4)	0.4336 (4)	1.0168 (2)	0.0658 (8)	
H4A	1.3424	0.5014	1.0488	0.079*	
C5	1.1795 (3)	0.4575 (3)	0.9320 (2)	0.0524 (7)	
H5A	1.1958	0.5429	0.9071	0.063*	
C6	1.0681 (3)	0.3567 (3)	0.88285 (18)	0.0424 (6)	
C7	0.9818 (3)	0.3853 (3)	0.79290 (18)	0.0432 (6)	
H7A	0.9854	0.4772	0.7771	0.052*	
C8	0.7473 (3)	0.2195 (2)	0.5826 (2)	0.0438 (6)	
C9	0.6441 (3)	0.2671 (2)	0.50316 (18)	0.0392 (6)	
C10	0.6698 (3)	0.3655 (2)	0.44024 (17)	0.0365 (5)	
C11	0.8216 (3)	0.4475 (3)	0.4339 (2)	0.0482 (6)	
H11A	0.8974	0.4238	0.4837	0.072*	
H11B	0.8035	0.5479	0.4493	0.072*	
H11C	0.8626	0.4242	0.3644	0.072*	
C12	0.5355 (3)	0.3773 (2)	0.37593 (16)	0.0348 (5)	
C13	0.5400 (3)	0.4737 (3)	0.29632 (19)	0.0482 (6)	
H13A	0.4382	0.4681	0.2607	0.072*	
H13B	0.6210	0.4448	0.2464	0.072*	
H13C	0.5636	0.5702	0.3306	0.072*	
C14	0.4126 (3)	0.2878 (2)	0.39320 (17)	0.0356 (5)	
C15	0.2514 (3)	0.2808 (2)	0.34768 (17)	0.0364 (5)	
C16	−0.0755 (3)	0.0451 (2)	0.31184 (19)	0.0454 (6)	
H16A	−0.0433	−0.0200	0.3532	0.055*	
C17	−0.2275 (3)	0.0243 (3)	0.25834 (19)	0.0452 (6)	
C18	−0.2791 (3)	0.1158 (3)	0.1937 (2)	0.0490 (7)	
C19	−0.4226 (4)	0.0886 (4)	0.1412 (2)	0.0686 (9)	
H19A	−0.4569	0.1500	0.0975	0.082*	
C20	−0.5150 (4)	−0.0277 (4)	0.1529 (3)	0.0798 (11)	
H20A	−0.6133	−0.0446	0.1179	0.096*	
C21	−0.4657 (4)	−0.1201 (4)	0.2151 (3)	0.0795 (10)	
H21A	−0.5291	−0.2005	0.2218	0.095*	
C22	−0.3236 (4)	−0.0936 (3)	0.2669 (2)	0.0639 (8)	
H22A	−0.2900	−0.1568	0.3094	0.077*	
O1	0.9338 (3)	0.13019 (19)	0.88189 (15)	0.0640 (6)	
H1	0.8908	0.1536	0.8305	0.096*	
O2	0.7611 (2)	0.09444 (16)	0.58491 (16)	0.0683 (6)	
O3	0.1986 (2)	0.38046 (15)	0.31228 (12)	0.0443 (4)	
O4	−0.1926 (2)	0.2321 (2)	0.17967 (17)	0.0681 (6)	
H4B	−0.1073	0.2355	0.2119	0.102*	
S1	0.46125 (8)	0.18434 (6)	0.48365 (5)	0.0450 (2)	

### Atomic displacement parameters (Å^2^)

**Table:**

	*U*^11^	*U*^22^	*U*^33^	*U*^12^	*U*^13^	*U*^23^
N1	0.0422 (13)	0.0467 (11)	0.0497 (12)	0.0023 (10)	−0.0194 (10)	0.0140 (9)
N2	0.0483 (13)	0.0348 (10)	0.0563 (13)	−0.0007 (9)	−0.0264 (11)	0.0116 (9)
N3	0.0329 (11)	0.0371 (10)	0.0555 (13)	0.0001 (9)	−0.0143 (10)	0.0054 (9)
N4	0.0351 (12)	0.0350 (10)	0.0654 (14)	−0.0012 (9)	−0.0191 (10)	0.0139 (9)
C1	0.0444 (16)	0.0562 (15)	0.0425 (14)	0.0076 (13)	−0.0018 (12)	0.0105 (12)
C2	0.068 (2)	0.0745 (19)	0.0507 (17)	0.0153 (17)	−0.0077 (15)	0.0200 (15)
C3	0.066 (2)	0.100 (2)	0.0455 (17)	0.022 (2)	−0.0139 (15)	0.0112 (17)
C4	0.0513 (18)	0.090 (2)	0.0512 (17)	0.0001 (17)	−0.0179 (14)	−0.0003 (16)
C5	0.0446 (16)	0.0620 (16)	0.0495 (15)	−0.0029 (13)	−0.0072 (13)	0.0081 (13)
C6	0.0372 (14)	0.0525 (14)	0.0378 (13)	0.0056 (12)	−0.0054 (11)	0.0087 (11)
C7	0.0417 (15)	0.0443 (13)	0.0445 (14)	0.0030 (12)	−0.0087 (12)	0.0103 (11)
C8	0.0358 (14)	0.0387 (12)	0.0574 (15)	−0.0030 (11)	−0.0172 (12)	0.0126 (11)
C9	0.0329 (13)	0.0339 (11)	0.0493 (14)	−0.0015 (10)	−0.0148 (11)	0.0053 (10)
C10	0.0332 (13)	0.0350 (11)	0.0390 (13)	0.0008 (10)	−0.0045 (10)	0.0007 (10)
C11	0.0361 (14)	0.0584 (15)	0.0488 (15)	−0.0058 (12)	−0.0059 (12)	0.0083 (12)
C12	0.0338 (13)	0.0354 (11)	0.0338 (12)	0.0009 (10)	−0.0044 (10)	0.0031 (9)
C13	0.0444 (15)	0.0530 (14)	0.0494 (15)	−0.0021 (12)	−0.0073 (12)	0.0169 (12)
C14	0.0347 (13)	0.0327 (11)	0.0390 (13)	0.0016 (10)	−0.0090 (10)	0.0057 (9)
C15	0.0369 (13)	0.0328 (11)	0.0380 (12)	0.0016 (10)	−0.0082 (10)	0.0028 (10)
C16	0.0406 (15)	0.0412 (13)	0.0547 (15)	−0.0008 (12)	−0.0119 (12)	0.0107 (11)
C17	0.0340 (14)	0.0495 (14)	0.0494 (15)	−0.0022 (12)	−0.0052 (12)	0.0028 (12)
C18	0.0382 (15)	0.0529 (15)	0.0542 (16)	0.0047 (13)	−0.0055 (12)	0.0039 (12)
C19	0.0429 (18)	0.091 (2)	0.069 (2)	0.0100 (18)	−0.0190 (15)	0.0056 (17)
C20	0.0352 (17)	0.114 (3)	0.079 (2)	−0.0092 (19)	−0.0133 (16)	−0.011 (2)
C21	0.050 (2)	0.096 (2)	0.086 (2)	−0.0327 (18)	−0.0060 (18)	0.008 (2)
C22	0.0540 (19)	0.0697 (18)	0.0668 (19)	−0.0183 (15)	−0.0059 (15)	0.0136 (15)
O1	0.0706 (15)	0.0566 (11)	0.0683 (14)	−0.0086 (11)	−0.0167 (11)	0.0253 (10)
O2	0.0728 (15)	0.0334 (9)	0.0996 (15)	−0.0046 (9)	−0.0467 (12)	0.0197 (9)
O3	0.0427 (10)	0.0366 (8)	0.0548 (10)	0.0001 (8)	−0.0179 (8)	0.0135 (7)
O4	0.0557 (13)	0.0601 (11)	0.0935 (16)	0.0018 (10)	−0.0228 (11)	0.0293 (11)
S1	0.0398 (4)	0.0391 (3)	0.0577 (4)	−0.0084 (3)	−0.0212 (3)	0.0167 (3)

### Geometric parameters (Å, º)

**Table:**

N1—C7	1.270 (3)	C11—H11A	0.9600
N1—N2	1.368 (3)	C11—H11B	0.9600
N2—C8	1.326 (3)	C11—H11C	0.9600
N2—H2	0.8600	C12—C14	1.355 (3)
N3—C16	1.261 (3)	C12—C13	1.491 (3)
N3—N4	1.365 (3)	C13—H13A	0.9600
N4—C15	1.331 (3)	C13—H13B	0.9600
N4—H4	0.8600	C13—H13C	0.9600
C1—O1	1.337 (3)	C14—C15	1.462 (3)
C1—C2	1.368 (3)	C14—S1	1.708 (2)
C1—C6	1.391 (4)	C15—O3	1.217 (3)
C2—C3	1.354 (4)	C16—C17	1.432 (3)
C2—H2A	0.9300	C16—H16A	0.9300
C3—C4	1.370 (4)	C17—C22	1.375 (4)
C3—H3A	0.9300	C17—C18	1.384 (4)
C4—C5	1.364 (4)	C18—O4	1.338 (3)
C4—H4A	0.9300	C18—C19	1.367 (4)
C5—C6	1.377 (3)	C19—C20	1.355 (5)
C5—H5A	0.9300	C19—H19A	0.9300
C6—C7	1.433 (3)	C20—C21	1.361 (5)
C7—H7A	0.9300	C20—H20A	0.9300
C8—O2	1.205 (3)	C21—C22	1.354 (4)
C8—C9	1.476 (3)	C21—H21A	0.9300
C9—C10	1.355 (3)	C22—H22A	0.9300
C9—S1	1.690 (3)	O1—H1	0.8200
C10—C12	1.411 (3)	O4—H4B	0.8200
C10—C11	1.477 (4)		
			
C7—N1—N2	117.0 (2)	H11A—C11—H11C	109.5
C8—N2—N1	118.42 (19)	H11B—C11—H11C	109.5
C8—N2—H2	120.8	C14—C12—C10	111.9 (2)
N1—N2—H2	120.8	C14—C12—C13	126.7 (2)
C16—N3—N4	118.1 (2)	C10—C12—C13	121.3 (2)
C15—N4—N3	117.69 (19)	C12—C13—H13A	109.5
C15—N4—H4	121.2	C12—C13—H13B	109.5
N3—N4—H4	121.2	H13A—C13—H13B	109.5
O1—C1—C2	117.5 (3)	C12—C13—H13C	109.5
O1—C1—C6	122.1 (2)	H13A—C13—H13C	109.5
C2—C1—C6	120.4 (3)	H13B—C13—H13C	109.5
C3—C2—C1	119.6 (3)	C12—C14—C15	126.6 (2)
C3—C2—H2A	120.2	C12—C14—S1	112.18 (17)
C1—C2—H2A	120.2	C15—C14—S1	121.11 (18)
C2—C3—C4	121.3 (3)	O3—C15—N4	121.2 (2)
C2—C3—H3A	119.4	O3—C15—C14	121.7 (2)
C4—C3—H3A	119.4	N4—C15—C14	117.08 (19)
C5—C4—C3	119.3 (3)	N3—C16—C17	119.9 (2)
C5—C4—H4A	120.3	N3—C16—H16A	120.0
C3—C4—H4A	120.3	C17—C16—H16A	120.0
C4—C5—C6	120.9 (3)	C22—C17—C18	118.1 (3)
C4—C5—H5A	119.6	C22—C17—C16	119.6 (2)
C6—C5—H5A	119.6	C18—C17—C16	122.3 (2)
C5—C6—C1	118.5 (2)	O4—C18—C19	117.7 (3)
C5—C6—C7	119.0 (2)	O4—C18—C17	122.2 (2)
C1—C6—C7	122.6 (2)	C19—C18—C17	120.1 (3)
N1—C7—C6	120.5 (2)	C20—C19—C18	120.0 (3)
N1—C7—H7A	119.8	C20—C19—H19A	120.0
C6—C7—H7A	119.8	C18—C19—H19A	120.0
O2—C8—N2	123.2 (2)	C19—C20—C21	120.9 (3)
O2—C8—C9	121.3 (2)	C19—C20—H20A	119.6
N2—C8—C9	115.5 (2)	C21—C20—H20A	119.6
C10—C9—C8	131.5 (2)	C22—C21—C20	119.2 (3)
C10—C9—S1	112.63 (17)	C22—C21—H21A	120.4
C8—C9—S1	115.89 (19)	C20—C21—H21A	120.4
C9—C10—C12	112.1 (2)	C21—C22—C17	121.6 (3)
C9—C10—C11	125.2 (2)	C21—C22—H22A	119.2
C12—C10—C11	122.6 (2)	C17—C22—H22A	119.2
C10—C11—H11A	109.5	C1—O1—H1	109.5
C10—C11—H11B	109.5	C18—O4—H4B	109.5
H11A—C11—H11B	109.5	C9—S1—C14	91.09 (12)
C10—C11—H11C	109.5		
			
C7—N1—N2—C8	172.0 (2)	C11—C10—C12—C13	0.5 (3)
C16—N3—N4—C15	−173.2 (2)	C10—C12—C14—C15	174.1 (2)
O1—C1—C2—C3	−179.5 (3)	C13—C12—C14—C15	−9.0 (4)
C6—C1—C2—C3	−0.5 (4)	C10—C12—C14—S1	−2.3 (2)
C1—C2—C3—C4	1.1 (5)	C13—C12—C14—S1	174.64 (18)
C2—C3—C4—C5	−0.4 (5)	N3—N4—C15—O3	3.1 (3)
C3—C4—C5—C6	−1.1 (4)	N3—N4—C15—C14	−178.0 (2)
C4—C5—C6—C1	1.6 (4)	C12—C14—C15—O3	−20.8 (4)
C4—C5—C6—C7	−178.9 (2)	S1—C14—C15—O3	155.27 (18)
O1—C1—C6—C5	178.1 (2)	C12—C14—C15—N4	160.2 (2)
C2—C1—C6—C5	−0.9 (4)	S1—C14—C15—N4	−23.7 (3)
O1—C1—C6—C7	−1.3 (4)	N4—N3—C16—C17	−174.8 (2)
C2—C1—C6—C7	179.7 (2)	N3—C16—C17—C22	178.7 (2)
N2—N1—C7—C6	178.1 (2)	N3—C16—C17—C18	1.8 (4)
C5—C6—C7—N1	167.1 (2)	C22—C17—C18—O4	−179.2 (3)
C1—C6—C7—N1	−13.4 (4)	C16—C17—C18—O4	−2.2 (4)
N1—N2—C8—O2	−4.8 (4)	C22—C17—C18—C19	0.6 (4)
N1—N2—C8—C9	172.8 (2)	C16—C17—C18—C19	177.6 (2)
O2—C8—C9—C10	−133.2 (3)	O4—C18—C19—C20	−179.9 (3)
N2—C8—C9—C10	49.2 (4)	C17—C18—C19—C20	0.3 (4)
O2—C8—C9—S1	45.7 (3)	C18—C19—C20—C21	−1.1 (5)
N2—C8—C9—S1	−132.0 (2)	C19—C20—C21—C22	1.0 (5)
C8—C9—C10—C12	−179.5 (2)	C20—C21—C22—C17	0.0 (5)
S1—C9—C10—C12	1.6 (2)	C18—C17—C22—C21	−0.8 (4)
C8—C9—C10—C11	3.4 (4)	C16—C17—C22—C21	−177.8 (3)
S1—C9—C10—C11	−175.46 (18)	C10—C9—S1—C14	−2.47 (18)
C9—C10—C12—C14	0.4 (3)	C8—C9—S1—C14	178.49 (18)
C11—C10—C12—C14	177.6 (2)	C12—C14—S1—C9	2.72 (17)
C9—C10—C12—C13	−176.7 (2)	C15—C14—S1—C9	−173.90 (18)

### Hydrogen-bond geometry (Å, º)

**Table:**

*D*—H···*A*	*D*—H	H···*A*	*D*···*A*	*D*—H···*A*
O1—H1···N1	0.82	1.91	2.626 (4)	145
N2—H2···O3^i^	0.86	1.97	2.789 (4)	159
N4—H4···O2^ii^	0.86	1.97	2.812 (4)	165
O4—H4*B*···N3	0.82	1.85	2.569 (4)	146

Symmetry codes: (i) −*x*+1, −*y*+1, −*z*+1; (ii) −*x*+1, −*y*, −*z*+1.
